# Signaling pathways governing the pathobiological features and antifungal drug resistance of *Candida auris*

**DOI:** 10.1128/mbio.02475-23

**Published:** 2025-04-03

**Authors:** Hyunjin Cha, Doyeon Won, Yong-Sun Bahn

**Affiliations:** 1 Department of Biotechnology, College of Life Science and Biotechnology, Yonsei University, Seoul, Republic of Korea; Instituto Carlos Chagas, Curitiba, Brazil

**Keywords:** *C. auris*, signaling pathways, virulence, antifungal drug resistance, candidiasis

## Abstract

*Candida auris* is an emerging multidrug-resistant fungal pathogen that poses a significant global health threat. Since its discovery in 2009, *C. auris* has rapidly spread worldwide, causing severe infections with high mortality rates, particularly in healthcare settings. Its ability to persist in the environment, form biofilms, and resist multiple antifungal drugs underscores the urgent need to understand its pathogenicity mechanisms and associated signaling pathways. Such insights are crucial for elucidating its unique virulence traits and developing targeted therapeutic strategies. Current studies have identified several key pathways involved in its pathogenicity and antifungal drug resistance. The Ras/cAMP/PKA pathway regulates critical virulence factors, including thermotolerance, morphological plasticity, and biofilm formation. The mitogen-activated protein kinase (MAPK) and calcineurin pathways contribute to stress responses and antifungal drug resistance. The regulation of Ace2 and morphogenesis (RAM) pathway influences cell aggregation, while the target of rapamycin (TOR) pathway affects filamentous growth and biofilm development. However, the distinct characteristics of *C. auris*, such as its rapid environmental spread and clade-specific traits, warrant further investigation into additional signaling pathways. This review provides a comprehensive analysis of known signaling pathways associated with *C. auris* pathogenicity and antifungal drug resistance, integrating insights from other fungal pathogens. By synthesizing current knowledge and identifying research gaps, this review offers new perspectives on future research directions and potential therapeutic targets against this formidable pathogen.

## INTRODUCTION

The global incidence of diseases caused by fungal pathogens is rising, posing an increasing public health threat. A recent comprehensive analysis of fungal infection prevalence and mortality rates across more than 120 countries revealed that over 6.55 million people suffer from life-threatening fungal diseases, leading to more than 3.75 million deaths annually (1). Notably, *Candida* species infections are identified as the most prevalent fungal pathogens. Each year, over 1,565,000 individuals are affected by invasive candidiasis or bloodstream infections caused by *Candida*, resulting in approximately 995,000 deaths. This accounts for 63.6% of the total fatalities from fungal infections, highlighting a critical public health concern (1).

*Candida auris* was first reported in 2009 from a patient’s ear canal in Japan, with its name *auris* derived from the Latin word for ear (2). However, retrospective studies indicate that *C. auris* was present in South Korea as early as 1996 (3), predating its first reported case in Japan. *C. auris* is one of the *Candida* species responsible for invasive candidiasis and has established itself as a major pathogenic *Candida* species, accounting for 26% of candidemia cases in South Africa and nearly 40% in India (4, 5).

Currently, *C. auris* is rapidly spreading worldwide, with infection cases reported across most continents and increasing at an alarming rate (6). The Centers for Disease Control and Prevention (CDC) reported a 95% increase in annual clinical cases of *C. auris* infections in the United States in 2021 (7). Initially confined to metropolitan areas, *C. auris* is now detected in more than half of the regions in the United States (7). Notably, *C. auris* exhibits varying fatality rates depending on the geographic region, ranging from 30% to 60% (8). Given its rapid transmission, its ability to cause candidemia, and its relatively high fatality rate, urgent measures are necessary to mitigate the threat posed by this emerging fungal pathogen.

*C. auris* has garnered significant attention due to its notable characteristics, including thermotolerance and resistance to multiple drugs, both of which have contributed to high fatality rates in human infections. However, despite *C. auris* possessing traits that make it particularly challenging to control, the reasons behind its recent emergence as a human pathogen remain unclear. The origin and emergence of *C. auris* have yet to be fully elucidated. Among the proposed hypotheses, one noteworthy suggestion is that global warming has facilitated its emergence (9). This hypothesis suggests that *C. auris* evolved into a human pathogen through natural selection, favoring thermotolerant strains potentially driven by global warming caused by human activity (9). Specifically, it proposes that rising temperatures in wetland ecosystems, induced by global warming, created environmental conditions that favored the selection of thermotolerant species such as *C. auris*, contributing to its emergence as a human pathogen (9). However, as the exact mechanisms underlying the emergence and spread of *C. auris* remain unresolved, further research in this area is urgently needed.

*C. auris* colonizes the skin, leading to infections and subsequent transmission, especially within healthcare facilities. Data from the PINC-A1 Healthcare Database (PHD), which covers over 1,000 hospitals across the United States (10), identified 192 hospitalizations related to *C. auris* between 2017 and 2022 (10). *C. auris* has a notable capacity for rapid environmental contamination, as evidenced by its detection on 33.2% of room surfaces prior to disinfection (11). Even after disinfection, residual *C. auris* contamination persisted on 20.5% of room surfaces after 4 h (11). Given its rapid and widespread environmental dissemination, implementing regular and frequent disinfection protocols is crucial.

*C. auris* belongs to the CTG clade of *Candida* species, which translates the CUG codon into serine instead of leucine, similar to *Candida albicans*, *Candida tropicalis*, and *Candida parapsilosis* (12). Analysis of *C. auris* isolates from various regions has revealed four primary clades based on geographic origin (13): clade I (South Asian), clade II (East Asian), clade III (African), and clade IV (South American) (13–16). In 2018, a strain isolated in Iran suggested the potential emergence of a fifth major clade (15, 16). Sequencing analysis of clade I through IV has confirmed that *C. auris* has a genome size ranging from 12.1 Mb to 12.7 Mb, distributed across seven chromosomes (13, 17). The number of protein-coding genes varies slightly between clades, ranging from 5,400 to 5,600 (13, 17). Similar to other *Candida* species, *C. auris* possesses a mating-type locus, indicating the potential for mating. Sequencing revealed that clade I and IV have the *MTL*α type, while clade II and III carry the *MTL***a** type (13, 14, 17, 18). Genetically distinct isolates of *C. auris* recently identified in Bangladesh and Singapore have led to the proposal of a novel sixth clade, expanding our understanding of the species’ genetic diversity (19, 20).

Antifungal drug resistance presents a significant challenge in the treatment of invasive candidiasis. *C. auris* is a pan-drug-resistant fungal pathogen exhibiting resistance to several antifungal drugs commonly used for fungal infections, including azoles, amphotericin B (AMB), and echinocandins. The CDC’s Antimicrobial Resistance Laboratory (AR Lab) Network tested 1,294 *C*. *auris* isolates in 2020, finding that 86% were resistant to azoles and 26% were resistant to AMB (7). Although echinocandin resistance remains relatively low, there is a concerning rise in the number of isolates resistant to multiple drug classes (7). Given the increasing severity of *C. auris* infections and its multidrug-resistant nature, it is crucial to understand the factors contributing to its pathogenicity, transmission, and antifungal drug resistance, along with the underlying mechanisms. This review focuses on the virulence traits of *C. auris*, its mechanisms of antifungal drug resistance, and the associated signaling pathways, offering valuable insights for developing novel therapeutic strategies against this formidable fungal pathogen.

## SIGNALING PATHWAYS FOR HOST TEMPERATURE ADAPTATION AND THERMOTOLERANCE

Adjusting to the host’s temperature is crucial for a fungus to become a human pathogen. *C. auris* thrives optimally at temperatures of 30°C and 37°C and exhibits thermotolerance, meaning it can endure higher temperatures of up to 42°C (21). This ability to tolerate higher temperatures is associated with the emergence of *C. auris* as a human pathogen. Genetically distinct clades of *C. auris* appeared independently across three continents, leading to the belief that *C. auris* was initially an environmental fungus before it emerged as a human pathogen (14). Additionally, the ability to survive at high temperatures enables *C. auris* to colonize animals, such as birds, which have elevated body temperatures facilitating its spread to distant regions (9).

In eukaryotic cells, the heat shock response involves various heat shock proteins (HSPs), which play a critical role in enhancing protein folding and degrading damaged proteins (22). In *C. albicans*, the phosphorylation of the heat shock transcription factor (Hsf1) in response to heat stress triggers the transcriptional activation of HSP genes through binding to the heat shock element (HSE) (23). Heat shock protein 90 (Hsp90) subsequently downregulates Hsf1, completing a regulatory feedback loop (23). In addition to the immediate response, elevated temperatures lead to long-term cell wall remodeling to cope with heat stress, a process mediated by mitogen-activated protein kinase (MAPK) pathways in *C. albicans* (23). Furthermore, the cyclic AMP (cAMP)/protein kinase A (PKA) pathway is crucial for adapting to heat shock in both the model fungus *Saccharomyces cerevisiae* and *C. albicans* (24). The calcium-calcineurin pathway is involved in thermotolerance in *Candida glabrata* and *Cryptococcus neoformans* (24).

**Fig 1:**
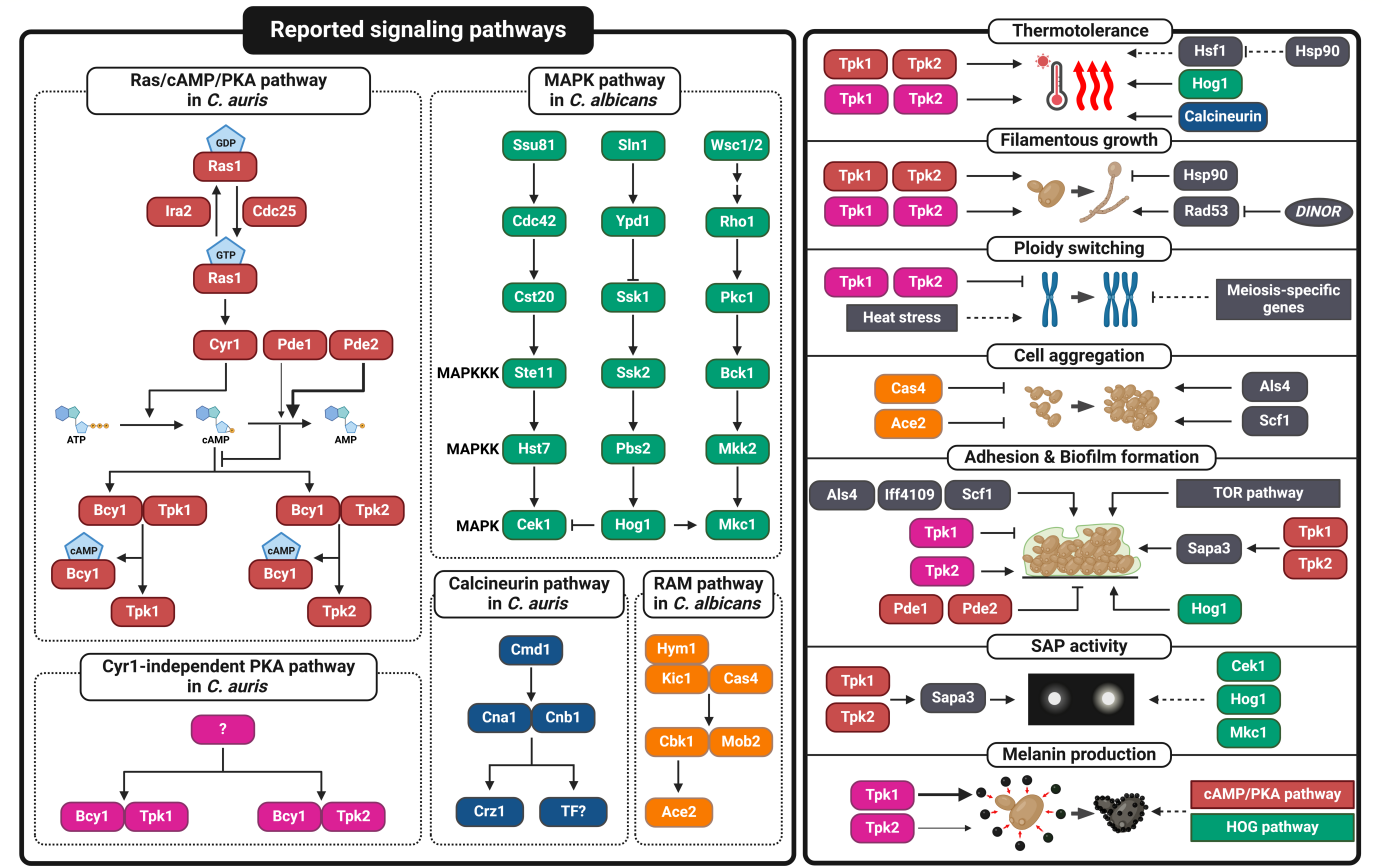
Overview of signaling pathways and mechanisms governing the virulence traits of *Candida auris*. This figure provides a summary of the signaling pathways and mechanisms that regulate the virulence traits of *C. auris*. The left panel highlights key signaling pathways, including the Ras/cAMP/PKA pathway (red), Cyr1-independent PKA pathway (deep pink), MAPK pathways (green), calcineurin pathway (dark blue), and RAM pathway (orange). These pathways feature components identified in either *C. auris* or *C. albicans*. Among them, several pathways characterized in *C. albicans* involve components that perform analogous functions in *C. auris* although the complete pathways in *C. auris* remain to be fully elucidated. The right panel categorizes key virulence traits of *C. auris* and their corresponding pathway regulators. Dashed arrows represent interactions identified in other fungal species but not yet confirmed in *C. auris*. Components not included in the left panel are shown in gray. This figure was created using BioRender (https://biorender.com/).

Basal growth and thermotolerance in *C. auris* B8441 (AR0387, clade I, isolated from Pakistan) are regulated by the cAMP/PKA signaling pathway (Table 1) (25). This pathway is an evolutionarily conserved signaling cascade that plays a critical role in the pathogenicity of various fungal pathogens (26). The core components of the *C. auris* cAMP/PKA pathway include adenylyl cyclase Cyr1, which converts ATP into cAMP, and PKA, which consists of two catalytic subunits (Tpk1 and Tpk2) and a single regulatory subunit (Bcy1) (25). Once cAMP is produced, it binds to the PKA regulatory subunit, releasing and activating the PKA catalytic subunits (Fig. 1). Typically, fungal adenylyl cyclases are activated by either RAS proteins or G-protein coupled receptors (GPCRs) associated with heterotrimer G protein complex (Gαβγ), or a combination of these (27). However, in *C. auris*, adenylyl cyclase contains only the RAS-binding domain and is primarily activated by Ras1, not by the GPCR Gpr1 and Gα subunit Gpa2 (Fig. 1) (27). Inactivation of the cAMP pathway markedly reduces the growth of *C. auris* across different temperatures (25). Interestingly, the deletion of both *TPK1* and *TPK2* causes more severe growth defects than the deletion of *CYR1*, indicating that PKA could have both Cyr1-dependent and independent roles in growth and thermotolerance (25).

**TABLE 1:** Different clades and strains of *C. auris* mentioned in this review

Clade	Strain	Origin	Studied signaling pathways/phenotypes
**I**	B8441(AR0387)	Pakistan	– Ras/cAMP/PKA1 pathway (25, 27) – Calcineurin pathway (28) – Scf1 (adhesin) (29) – Sapa3 (secreted aspartyl protease) (30)
	B11109 (AR0382)	Pakistan	– Iff4109 (adhesin) (29) – Scf1 (adhesin) (29) – RAM pathway (31, 32)
	B11203 (AR0389)	India	– Hog1 MAPK pathway (33)
	CBS12766	India	– *DINOR* (virulence and stress response) (34)
	BJCA001	China	– Als4 (adhesin) (35)
**II**	CBS10913	Japan	– *DINOR* (virulence factor and stress regulator) (34) – TOR pathway (36)
	B11220 (AR0381)	Japan	– Cna1 (calcineurin catalytic subunit) (28) – RAM pathway (31, 32)
**III**	B11221 (AR0383)	South Africa	– Cna1 (calcineurin catalytic subunit) (28) – Aggregation phenotypes (37)
	B11222 (AR0384)	South Africa	– Hog1 MAPK pathway (33)
**IV**	B11244 (AR0385)	Venezuela	– Aggregation phenotypes (37)
	B11245 (AR0386)	Venezuela	– Cna1 (calcineurin catalytic subunit) (28)

Hyperactivation of the cAMP/PKA pathway does not affect basal growth but inhibits thermotolerance at temperatures higher than 42°C (27). For the negative feedback regulation of the cAMP pathway, *C. auris* utilizes both low- and high-affinity phosphodiesterases, Pde1 and Pde2, respectively (Fig. 1). Deletion of *PDE2* significantly reduces thermotolerance and increases the expression of HSPs (27). Surprisingly, inactivation of the cAMP/PKA pathway (e.g., *cyr1*Δ, *tpk1*Δ *tpk2*Δ, and *ras1*Δ) does not influence the virulence of *C. auris* in a systemic infection model (25, 27). In contrast, hyperactivation of the cAMP/PKA pathway (e.g., *bcy1*Δ and *pde2*Δ) attenuates the systemic virulence of *C. auris* (25, 27). Notably, growth at the host’s physiological temperature of 37°C is not significantly affected in the hyperactive cAMP mutants, suggesting that their role in thermotolerance may not contribute to virulence. Instead, these hyperactivated cAMP mutants exhibit severe growth defects under nutrient-starved conditions within the host, which may lead to their reduced virulence (25, 27).

In addition to the cAMP/PKA pathway, the high osmolarity glycerol (HOG) and calcineurin pathways also contribute to thermotolerance in *C. auris* (Fig. 1). The HOG pathway is mediated by the Hog1 MAPK, a stress-activated protein kinase that responds to various environmental stresses, including heat stress (24). The *hog1*∆ mutants in both *C. auris* B11222 strain (AR0384, clade III, isolated from South Africa) and B11203 strain (AR0389, clade I, isolated from India) exhibit a growth defect at 42°C, indicating that the Hog1 MAPK pathway is involved in thermotolerance in *C. auris* (Table 1) (33). The calcineurin pathway is a conserved signaling cascade that regulates cellular stress responses in fungal species (38). Calcineurin is a serine/threonine protein phosphatase composed of two subunits: a catalytic subunit (Cna1) and a regulatory subunit (Cnb1) (38). In *C. albicans*, the deletion of *CMP1* (*CNA1*) does not affect growth at elevated temperatures (39). However, calcineurin plays a crucial role in thermotolerance in other major fungal species, including *C. glabrata* and *C. neoformans* (40, 41). Similar to *C. albicans*, the calcineurin pathway in *C. auris* B8441 does not appear to influence growth at temperatures between 37°C and 42°C (Table 1) (28). Calcineurin is required for growth at higher temperatures exceeding 43°C, suggesting that it plays a role in extreme thermotolerance (28). While the ability to adapt to host physiological temperatures is considered a common virulence trait among most human fungal pathogens, the specific role of thermotolerance in *C. auris* virulence across different clades requires further investigation.

## SIGNALING PATHWAYS FOR MORPHOLOGICAL DEVELOPMENT AND PLOIDY SWITCHING

The phenotypic plasticity of fungal pathogens can offer a significant advantage in the host environment. *C. auris* displays a range of morphological diversity, transitioning from yeast to filamentous forms (42), and even exhibiting pseudohyphal growth under genotoxic stress (43). One of the most well-studied pathways related to morphogenesis in *C. auris* is the Ras/cAMP/PKA pathway. The PKA catalytic subunits, Tpk1 and Tpk2, regulate filamentation both in a Cyr1-dependent and -independent manner (Fig. 1) (25, 27). Notably, high-temperature stress activates Hsp90, which represses filamentous growth in *C. auris* (Fig. 1) (44). However, it is unclear whether this repression occurs via interaction with the leucine-rich domain of Cyr1, as observed in *C. albicans* (45). Since this domain is also present in the Cyr1 protein of *C. auris* (27), further investigation is needed to determine the nature of this interaction.

Deletion of the DNA damage-inducible non-coding RNA (*DINOR*) induces filamentous growth in *C. auris* CBS10913 (clade II, isolated from Japan) and CBS12766 (clade I, isolated from India) strains (Table 1) (34). DNA damage resulting from *DINOR* deficiency activates Rad53, and the Rad53-mediated DNA damage response triggers filamentation (Fig. 1) (34). Since one of the genes that genetically interacts with *DINOR* is linked to the target of rapamycin (TOR) signaling pathway (34), it is crucial to further investigate the interconnected roles of *DINOR* and TOR signaling pathway in filamentation.

In addition to morphological transition, *C. auris* undergoes ploidy switching from haploid to diploid to better adapt to environmental conditions (46, 47). Tpk1 and Tpk2 suppress this ploidy switching independently of Cyr1 (Fig. 1) (25). Given the potential for other upstream regulators to activate the PKA complex, further research into these factors is necessary. Since heat stress is known to induce a ploidy shift in *C. albicans* (48), it would be essential to explore whether heat stress similarly triggers ploidy switching in *C. auris* and whether the PKA complex mediates this process (Fig. 1). Recent studies have identified meiosis-specific genes, such as *DMC1* and *REC8* in *C. neoformans* and *SPO11* in *C. albicans*, as key players in ploidy reduction (49). Therefore, investigating the relationship between meiosis-specific genes and ploidy switching in *C. auris* would provide valuable insights (Fig. 1).

Another major morphological feature of *C. auris* is its ability to form aggregates. This characteristic is clade-specific; in Sabouraud dextrose broth, strains from clades III (B11221, AR0383, isolated from South Africa) and IV (B11244, AR0385, isolated from Venezuela) form aggregates, whereas strains from clades I and II do not (Table 1) (37). *C. auris* displays two distinct types of aggregative morphology. One type arises from defects in cell division, while the other is due to the overexpression of genes related to adhesion (35, 37). In the first type, aggregation occurs when the regulation of Ace2 and morphogenesis (RAM) pathway is dysfunctional in *C. auris* B11220 (AR0381, clade II, isolated from Japan) and B11109 (AR0382, clade I, isolated from Pakistan) strains (Table 1) (31, 32). Deletion of *CAS4* (*TAO3*) or *ACE2*, which are components of the RAM pathway, has been shown to induce aggregation (Fig. 1) (31, 32). This form of aggregation may result from microevolutionary changes to enhance survival in the host environment during systemic infections (32). The second type of aggregation is caused by the upregulation of agglutinin-like sequence 4 (*ALS4*), a gene encoding cell wall adhesin, in *C. auris* BJCA001 (clade III, isolated from China) (35), or surface colonization factor 1 (*SCF1*) in the B8441 and B11109 strains (Fig. 1) (Table 1) (50). This type of aggregation is unique in that it can revert to a unicellular form when treated with proteinase K or trypsin (35, 37). As these two types of aggregation differ in their biofilm formation abilities (35, 51–53), further research is needed to understand the distinctions between them.

## SIGNALING PATHWAYS FOR ADHESION AND BIOFILM FORMATION

A key factor contributing to the pathogenicity of *C. auris* is its ability to form biofilms, a feature also observed in many other pathogenic fungi, especially *C. albicans*. Biofilms differ from aggregations in that they involve the irreversible attachment to a surface, followed by the formation of a structured matrix composed of extracellular polymers (54). The biofilm formation process begins with the adhesion of planktonic cells to a solid surface, which is then followed by biofilm maturation (27, 54). After maturation, cell dispersion occurs, allowing new biofilms to form on other surfaces (27, 54). Therefore, the ability of *C. auris* to adhere to surfaces is a critical factor in biofilm development and persistence.

In *C. auris*, the prominent adhesins Als4 and IPF Family F 4109 (Iff4109) both play critical roles in surface settlement and biofilm formation in *C. auris* BJCA001 and B11109 strains, respectively (Fig. 1) (Table 1) (29, 35). Additionally, a *C. auris*-specific adhesin, Scf1, contributes to skin adhesion and biofilm formation in the B8441 and B11109 strains (Fig. 1) (29, 50). Unlike other adhesins that mediate attachment interactions through canonical hydrophobic interactions, Scf1 employs noncanonical-substrate interactions and is also required for biofilm formation (29).

The Ras/cAMP/PKA pathway is a key regulator of adhesion in *C. auris*, with its hyperactivation leading to increased expression of adhesion-related genes such as *ALS4* and *SIT1*, thereby enhancing adhesion (27). Another signaling cascade involved in adhesin regulation is the Hog1 MAPK pathway (55). In *HOG1* deletion mutants, the expression levels of *ALS4* and *SCF1* remain unchanged, whereas *IFF4109* expression is reduced (55). This suggests that the Hog1 MAPK pathway differentially regulates adhesin expression, influencing adhesion dynamics. Therefore, a comprehensive understanding of *C. auris* adhesin regulation requires elucidation of the interplay between multiple signaling pathways. For instance, the TOR signaling pathway has been implicated in adhesin gene regulation in *C. albicans* (56). This warrants investigation into its role, along with other adhesion-related pathways identified in *C. albicans* and related *Candida* species, within the context of *C. auris*.

The Ras/cAMP/PKA pathway is also involved in the biofilm formation of *C. auris*. Independently of Cyr1, Tpk1, and Tpk2 play opposing roles, with Tpk1 suppressing and Tpk2 promoting biofilm formation (Fig. 1) (25). Inhibiting the negative regulators of the cAMP/PKA pathway, Pde1 and Pde2, leads to hyperactivation of this pathway, resulting in increased expression of adhesion-related genes, which enhances biofilm formation (Fig. 1) (27).

The TOR signaling pathway contributes to biofilm formation in *C. auris* CBS10913 strain (Fig. 1) (Table 1). Treatment with TOR inhibitors like rapamycin or torin2 reduces biofilm formation and inhibits cell growth within the biofilm, leading to a decrease in biofilm biomass (36). Secreted aspartyl proteases in *C. auris* (Sapa), particularly Sapa3, are also involved in biofilm formation. Deletion of *SAPA3*, a key *SAP* gene in *C. auris*, significantly reduces biofilm formation in the B8441 strain (Table 1) (30). Since the expression of *SAPA3* is regulated by Tpk1 and Tpk2 of the Ras/cAMP/PKA pathway (30), these suggest that the PKA catalytic subunit may modulate biofilm formation through the regulation of *SAPA3* (Fig. 1). Additionally, the reduced biofilm formation observed in *HOG1* deletion strains suggests a potential link between the MAPK pathway and biofilm formation (Fig. 1) (55).

Recent research suggests that the correlation between aggregation and biofilm formation in *C. auris* may manifest in a clade- or strain-specific manner (35, 51–53). In studies infecting *Galleria mellonella* with clade I (non-aggregative form) and clade III (aggregative form) isolates, higher biofilm mass was observed in the non-aggregative morphology (51). Conversely, another study using identical isolates showed higher expression of biofilm-associated genes in the aggregative form (52). Further research using different clade III strains demonstrated that aggregation induced by *ALS4* amplification promotes biofilm formation (35, 37). Similar findings were observed in studies conducted in India using clinical isolates (53). These findings suggest a positive correlation between aggregative morphology and biofilm formation though clade-specific and strain-specific variations in *C. auris* are likely. Therefore, further research is necessary to fully understand these relationships.

## SIGNALING PATHWAYS FOR PRODUCING SECRETED ASPARTYL PROTEASES

The production of extracellular hydrolytic enzymes, such as SAP, is a key virulence factor in other fungal pathogens. In *C. albicans*, 10 *SAP* genes have been identified, and these proteases play a crucial role during colonization (57). Secreted proteases help fungal pathogens evade the host immune system by degrading host proteins, such as immunoglobulin A and antimicrobial peptides (58). Among the *SAP* genes in *C. albicans*, *SAP2* plays a pivotal role in SAP activity. Deletion of the *SAP2* gene in *C. albicans* results in a significant reduction in SAP activity and attenuated virulence (59). Several transcription factors and related signaling pathways regulate *SAP* expression in *C. albicans*. The MAPK and cAMP pathways regulate transcription factors Cph1 and Efg1, respectively, which control hyphal development and induce the expression of *SAP4*, *SAP5*, and *SAP6* (60). Conversely, transcription factors Nrg1 and Mig1, which target Tup1, negatively regulate the expression of *SAP* genes in *C. albicans* (60).

In *C. auris*, SAP activity varies across different isolates. BJCA001, the first isolate of *C. auris* in China, demonstrates variable SAP activity depending on its morphology and temperature (42, 61). Strains with the *MTL*α mating type from China show lower SAP activity than *MTL***a** strains (62). Interestingly, BJCA001 (*MTL***a** strain from China) exhibits higher SAP activity at 25°C but lower activity at 37°C compared to the *MTL*α strains from China (62). The cAMP/PKA pathway, mediated by Tpk1 and Tpk2, also regulates SAP activity in the *C. auris* B8441 strain (Table 1) (30). Specifically, Sapa3 (B9J08_004629) plays a pivotal role in SAP activity through the Ras/cAMP/PKA signaling pathway (Fig. 1) (30). Further research is needed to identify the transcription factors regulating SAP activity and to explore whether the MAPKs (*HOG1*, *CEK1*, and *MKC1*) regulate Sapa3, thereby contributing to SAP activity in *C. auris* (Fig. 1).

## SIGNALING PATHWAYS FOR MELANIN PRODUCTION

Melanin is a biological pigment produced by many fungal pathogens, contributing to their pathogenesis in humans. Melanization provides various virulence-associated traits, such as protection from environmental stresses (e.g., oxidative stress and UV radiation), resistance to antifungal agents, and perturbed interactions with host cells (63). The signaling pathway related to melanization has been well studied in *C. neoformans*. Under nutrient starvation, four transcription factors—Hob1, Bzp4, Usv101, and Mbs1—regulate the expression of the laccase enzyme Lac1, which is involved in melanin synthesis, transport, and accumulation (64). These transcription factors are regulated by the cAMP/PKA and HOG signaling pathways (64).

In *C. albicans*, melanin is produced using DOPA as a substrate, but specific genes and signaling cascades related to melanization remain unknown (63). In *C. auris*, melanization has been identified as a novel mechanism compared to other fungal pathogens (65). *C. auris* alkalinizes the extracellular environment, which oxidizes L-DOPA into melanin, producing granule-like structures that attach to surfaces (65). Melanization in *C. auris* is controlled by the PKA catalytic subunits Tpk1 and Tpk2, with Tpk1 playing a major role in a Cyr1-independent manner (Fig. 1) (66). As *C. auris* exhibits unique melanization mechanisms, further research is necessary to uncover the underlying signaling pathways responsible for melanin production (Fig. 1).

## SIGNALING PATHWAYS GOVERNING ANTIFUNGAL DRUG RESISTANCE

**Fig 2:**
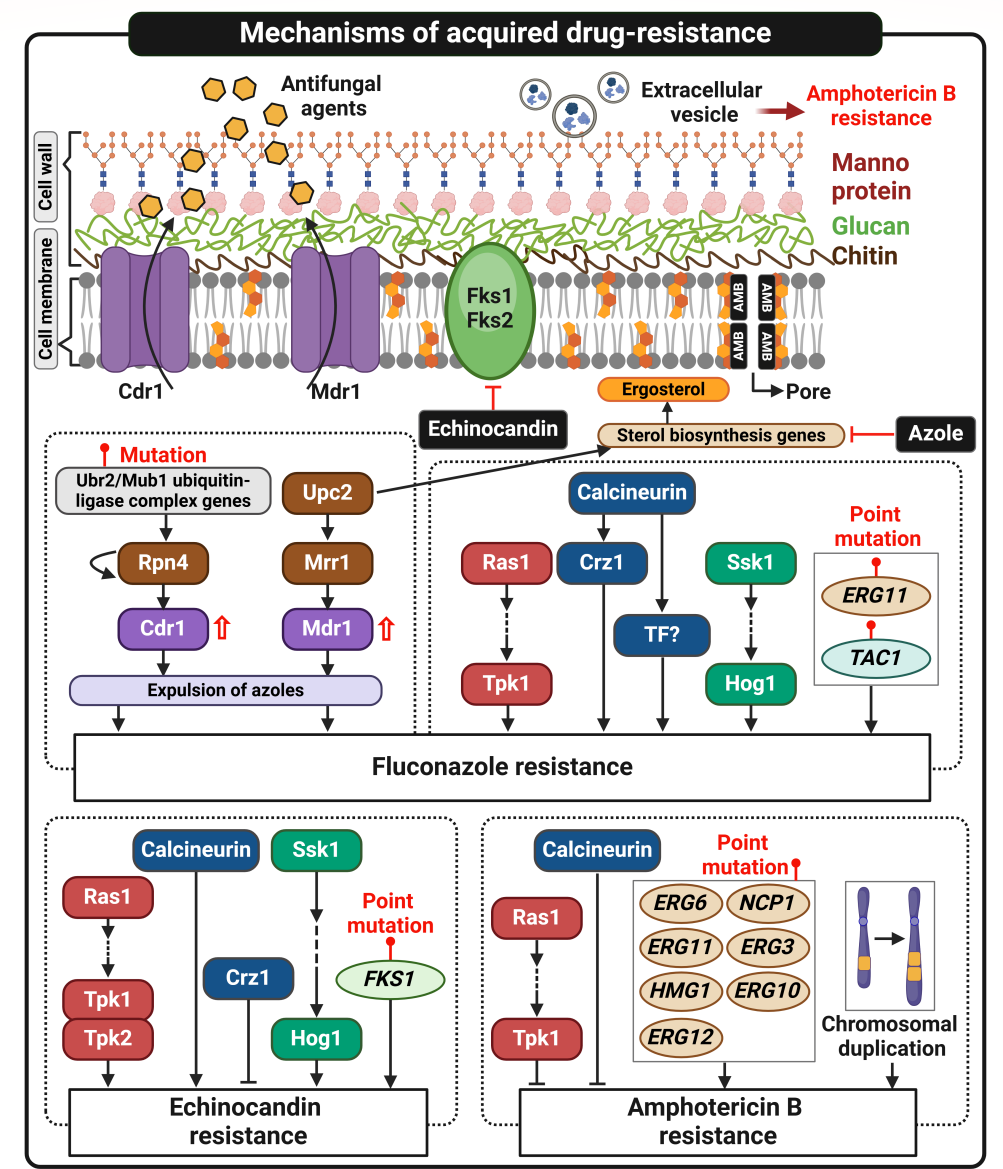
Overview of signaling pathways and mechanisms governing antifungal drug resistance in *Candida auris*. This figure highlights the primary mechanisms underlying antifungal resistance in *C. auris*. Azole resistance arises from point mutations in the *ERG11* or *TAC1* gene and the overexpression of efflux pumps, such as *CDR1*, which is regulated by transcription factors like Rpn4. Additional contributors include Mrr1 and Upc2, which influence efflux pump expression and ergosterol biosynthesis. Primary signaling pathways, including Ras/cAMP/PKA, calcineurin, and Hog1, also modulate azole resistance. Echinocandin resistance is primarily linked to mutations in *FKS1*, impairing β-1,3-glucan synthase. Calcineurin signaling plays a complex role in resistance mechanisms; while calcineurin promotes echinocandin resistance, its downstream factor Crz1 represses it, suggesting that additional calcineurin-dependent factors could be involved. Amphotericin B resistance is driven by mutations in sterol biosynthesis genes (e.g., *ERG6*, *ERG11*), chromosomal duplications, and extracellular vesicles (EVs). Notably, the Ras/cAMP/PKA and calcineurin pathways inhibit amphotericin B resistance. This figure was created using BioRender (https://biorender.com/).

With the increasing prevalence of multidrug-resistant *C. auris* isolates, it is crucial to understand signaling pathways involved in antifungal drug resistance in this pathogen. In other fungal species, azole resistance is primarily linked to mutations in the *ERG11* gene, which encodes lanosterol demethylase, the target of azole drugs (67, 68). The amino acid substitutions in the *ERG11* sequences of *C. auris* clinical isolates have been associated with fluconazole resistance (Fig. 2) (69).

In addition to mutations in *ERG11*, resistance can arise when the efflux pump *CDR1* is overexpressed, leading to the active expulsion of azole drugs from the cell (Fig. 2) (70). In *C. auris*, the transcription factors Tac1a and Tac1b, which are homologs of transcription factor Tac1 regulating the expression of *CDR1* in *C. albicans*, play a role in fluconazole resistance (70). However, mutations in *TAC1* are independent of *CDR1* expression in *C. auris* (Fig. 2) (70). Instead, the transcription activator Rpn4 plays a role in fluconazole resistance in *C. auris* by upregulating efflux pump genes like *CDR1*, thereby reducing the intracellular concentration of the drug (Fig. 2) (71). Rpn4 promotes its own expression through autoregulation by binding to a proteasome-associated control element (PACE) in its promoter (71). Additionally, mutations in the ubiquitin-ligase complex genes *UBR2* and *MUB1* stabilize Rpn4, which maintains high levels of efflux pump expression and increases resistance (Fig. 2) (71). Other factors, such as the transcription factor Mrr1 and the drug transporter Mdr1, also contribute to azole resistance in *C. auris* (72). This mechanism is not associated with *ERG11* mutations and *CDR1* upregulation (Fig. 2) (72). The transcription factor Upc2, which regulates the ergosterol biosynthesis pathway, contributes to azole resistance by controlling *ERG11* expression and activating the Mrr1/Mdr1 pathway (Fig. 2) (73).

In *C. albicans*, the cAMP/PKA pathway plays a key role in mediating azole resistance (74). Adenylate cyclases, encoded by *CDC35* in *C. albicans* and *CYR1* in *S. cerevisiae*, regulate azole resistance (74). Similarly, the cAMP/PKA pathway determines multidrug resistance in *C. auris*. Deletion of *CYR1* and *TPK1* affects susceptibility to drugs like fluconazole while conferring resistance to AMB, a pattern not observed in the *tpk2*∆ mutant (25). The mutants also exhibit altered expression of genes involved in ergosterol biosynthesis (*ERG11, ERG3*, and *ERG6*), suggesting that this pathway significantly influences ergosterol production, which in turn, impacts AMB resistance (25). The *ras1*∆ mutant shows enhanced azole susceptibility while displaying increased resistance to AMB (Fig. 2) (27).

Echinocandin resistance is primarily caused by mutations in the *FKS* gene, which encodes β-1,3-glucan synthase (Fig. 2) (75). This enzyme plays a critical role in synthesizing β-1,3-glucan, a key component of the fungal cell wall (75). The fungal cell wall is essential for maintaining cell shape and integrity, and it also facilitates adhesion and interaction with the host (75). Targeting β-1,3-glucan synthase with echinocandin drugs disrupts cell wall formation, leading to fungal cell death. Echinocandin-resistant *C. auris* isolates often have hot spot mutations in *FKS1* (Fig. 2) (76). These isolates exhibit a shared set of 362 differentially expressed genes, some of which are involved in cell wall function (76). Additionally, novel non-hot spot mutations downstream of hot spot 1 in *FKS1* have been identified, further contributing to echinocandin resistance (77). These findings suggest a diversity of *FKS1* mutations that drive echinocandin resistance in *C. auris* (77). In *C. auris*, the Ras/cAMP/PKA pathway is also involved in echinocandin resistance by affecting *FKS1* expression. *cyr1*∆ and *tpk1*∆ *tpk2*∆ mutants display increased susceptibility to caspofungin due to changes in *FKS1* expression (Fig. 2) (25). Similarly, the *ras1*∆ mutant shows enhanced echinocandin susceptibility (27).

The cell wall integrity (CWI) signaling pathway is also involved in resistance to azoles and echinocandins. Echinocandins inhibit the β-1,3-glucan synthesis, which leads to a chitin synthesis through protein kinase C (PKC)/CWI MAPK, calcineurin, and HOG pathways (78). In *C. auris*, mutants with deletions of *HOG1* and *SSK1* show significant susceptibility to antifungal drugs and cell wall stress agents (Fig. 2) (33). The calcium-calcineurin pathway is essential for azole and echinocandin resistance in *C. albicans* (79), and similarly, the calcineurin pathway plays a critical role in azole and echinocandin resistance in *C. auris* (Fig. 2) (28). Knockout of *CNA1*, which encodes the catalytic subunit of calcineurin, or *CNB1*, encoding the regulatory subunit, leads to a marked increase in susceptibility to various azole drugs, including fluconazole, posaconazole, and voriconazole (28). Moreover, the roles of calcineurin in azole resistance are conserved across different clades, including B11220 (AR0381, clade II, isolated from Japan), B11221 (AR0383, clade III, isolated from South Africa), and B11245 (AR0386, clade IV, isolated from Venezuela) (Table 1) (28). Deletion of *CRZ1*, a downstream transcription factor of calcineurin, also results in increased susceptibility, though to a lesser extent. Interestingly, while calcineurin is critical for echinocandin resistance, *crz1*Δ mutants display strong resistance to echinocandins, which is the opposite phenotype observed in the *cna1*Δ and *cnb1*Δ mutants (Fig. 2) (28). This suggests that other transcription factors may play a significant role in calcineurin-mediated resistance to echinocandins. Therefore, further investigation into additional signaling pathways and components is necessary to fully understand the mechanisms underlying drug resistance in *C. auris*, which may differ from those in other fungal species.

AMB is an antifungal drug commonly used to treat severe fungal infections, particularly when other antifungal agents are ineffective due to resistance (80). Its mechanism of action involves binding to ergosterol, a vital component of fungal cell membranes, forming pores that cause cell leakage and ultimately, cell death (Fig. 2) (81). AMB resistance in *C. auris* is driven by four major sterol alteration types, which are related to mutations in sterol biosynthesis genes (*ERG6*, *NCP1*, *ERG11*, *ERG3*, *HMG1*, *ERG10*, and *ERG12*) (Fig. 2) (82). Moreover, chromosomal duplications, particularly in chromosomes 4 and 6, were observed during resistance evolution, highlighting their role in acquired AMB resistance (Fig. 2) (82). In addition, extracellular vesicles (EVs) are known to play a role in AMB resistance (Fig. 2) (82). EVs extracted from a strain resistant to azoles but susceptible to AMB have been shown to enhance the survival rate of *C. auris* in a dose-dependent manner, increasing the minimum inhibitory concentration (MIC) of AMB by up to 16-fold (82). These EVs contain alcohol dehydrogenase 1 and homologs to *C. albicans* Mp65 (β-1,3-endoglucanase mannoprotein) and Xog1 (β-1,3-exoglucanase), which may contribute to drug resistance (82). As Xog1 regulates cell wall integrity via β-glucan modifications in *C. albicans*, its homolog in *C. auris* may similarly contribute to membrane repair and protection, potentially enhancing drug resistance (82). However, the specific signaling pathways involved in EVs-mediated AMB resistance remain unknown and warrant further investigation. Quantitative proteomic analysis of EVs, combined with large-scale forward and reverse genetic approaches to identify signaling components involved in EV formation, will provide deeper insights into these mechanisms.

## FORWARD AND REVERSE GENETIC TOOLS FOR STUDYING SIGNALING PATHWAYS IN *C. AURIS*

Comprehensive, high-throughput forward and reverse genetic approaches are fundamental for understanding gene function in *C. auris*. Forward genetics identifies causative genes based on specific phenotypes, while reverse genetics explores gene function through targeted manipulation. Forward genetics enables unbiased, genome-wide analyses, making it practically effective for phenotype-based studies. Advanced genetic tools, such as the piggyBac transposon system, have been employed to identify genes associated with pathogenicity, antifungal resistance, and environmental adaptation (83). Furthermore, *Agrobacterium tumefaciens*-mediated transformation, an insertional mutagenesis approach, provides an efficient method for genetic manipulation without requiring prior genetic engineering to introduce the transposon machinery (31). However, pinpointing target genes often requires additional steps, such as sequencing, to accurately identify genetic determinants.

In contrast, reverse genetics focuses on the roles of previously identified genes. Targeted gene transformation has been achieved using heat shock-based lithium acetate and electroporation methods, with the CRISPR-Cas9 system widely employed to improve transformation efficiency (3). This approach has facilitated the generation of knockout mutant libraries, advancing our understanding of complex signaling pathways. However, reverse genetics heavily relies on prior studies, which may overlook novel genes with significant biological roles. To address the limitations of both approaches, establishing large-scale knockout collections in *C. auris* through a combination of forward and reverse genetics would enable a more detailed exploration of signaling pathways and related mechanisms. Moreover, these methodologies are pivotal for advancing our understanding of *C. auris* virulence, thermotolerance, and unconventional virulence factor production pathways, providing critical insights that could inform future therapeutic strategies.

## FUTURE PERSPECTIVES

*C. auris* has emerged as a significant global health challenge due to its multidrug resistance, environmental adaptability, and rapid dissemination. Understanding the pathogenic mechanisms underlying *C. auris* is, therefore, of critical importance. However, compared to other comprehensively analyzed pathogenic fungi, research on *C. auris* remains limited owing to its relatively recent identification. To date, complete loss of virulence in systemic infection models has rarely been demonstrated in gene deletion mutants, and no definitive virulence factors, such as the capsule or melanin observed in *C. neoformans*, have been identified.

The persistence of virulence in cAMP/PKA pathway mutants, despite their severe growth defects and lack of thermotolerance, remains unexplained, suggesting the existence of cryptic virulence factors. Additionally, *C. auris* exhibits extreme thermotolerance, highlighting the importance of investigating the molecular mechanisms underlying this exceptional heat resistance. Identifying the unique processes that facilitate thermotolerance may uncover novel biological processes with therapeutic implications.

Furthermore, it is noteworthy that Sapa3, despite lacking a conventional signal sequence, plays a significant role in regulating SAP activity (30). However, the mechanisms underlying its secretion remain unclear, suggesting the possible involvement of non-canonical pathways such as extracellular vesicles or alternative signal sequences. Elucidating these mechanisms may not only provide novel therapeutic targets but also deepen our understanding of fungal secretion systems.
